# The Relationship Between Neuropsychiatric Symptoms, Nighttime Behaviors, and Alzheimer’s Disease CSF Biomarkers

**DOI:** 10.1093/geroni/igab046.2469

**Published:** 2021-12-17

**Authors:** Meina Zhang, Young-Eun Cho, Chooza Moon

**Affiliations:** 1 University of Iowa, Iowa City, Iowa, United States; 2 University of Iowa, university of Iowa, Iowa, United States

## Abstract

Alzheimer's disease (AD) commonly involves neuropsychiatric symptoms (NPS), such as nighttime behaviors (or sleep disturbance), hallucination, delusion, or mood changes. However, it is unclear how NPS and sleep disturbances are correlated with AD biomarkers. The purpose of this analysis was to examine how NPS and nighttime behaviors are associated with AD CSF biomarkers by cognitive status. A total of 1,667 subjects’ (mean age = 69.4 SD=9.3, 48 % (808) were male) data from the National Alzheimer’s Disease Coordinating Center (NACC) were used, including subjects with dementia (n = 577), mild cognitive impairment (MCI, n = 363), cognitive impairment but not MCI (n = 47), cognitive impairment due to Alzheimer’s etiology (n= 608), and normal cognition (n = 680). The nighttime symptoms, number, and severity of NPS were assessed using the Neuropsychiatric Inventory Questionnaire Quick Version (NPI-Q). Cerebrospinal fluid (CSF) samples were analyzed for Aβ42,dft5 t-tau, p-tau. We used generalized linear models to explore the associations accounting for age, sex, APOE4 alleles, and BMI. We found the number of NPS were associated with Aβ42 (p = 0.042) in individuals with MCI, impaired, or dementia due to Alzheimer’s etiology. Yet, the number of NPS were not associated with t-tau or p-tau in individuals with and without dementia. The severity of NPS including nighttime symptoms were not associated with biomarkers. Our results could suggest that the number of NPS can be reflected by higher CSF Aβ42 levels in the individuals with Alzheimer’s etiology. Future longitudinal analyses are warranted to understand the causal relationships.

